# Comparing the Flavor Characteristics of 71 Tomato (*Solanum lycopersicum*) Accessions in Central Shaanxi

**DOI:** 10.3389/fpls.2020.586834

**Published:** 2020-12-10

**Authors:** Guoting Cheng, Peipei Chang, Yuanbo Shen, Liting Wu, Ahmed H. El-Sappah, Fei Zhang, Yan Liang

**Affiliations:** ^1^College of Horticulture, Northwest A&F University, Yangling, China; ^2^State Key Laboratory of Crop Stress Biology in Arid Regions, Northwest A&F University, Yangling, China; ^3^Institute of Agricultural Sciences, Dezhou, China; ^4^Department of Genetics, Faculty of Agriculture, Zagazig University, Zagazig, Egypt

**Keywords:** taste compound, volatiles, hedonism score, odor activity value, DTOPSIS analysis

## Abstract

Flavor is an important quality of mature tomato fruits. Compared with heirloom tomatoes, modern commercial tomato cultivars are considerably less flavorful. This study aimed to compare the flavor of 71 tomato accessions (8 pink cherry, PC; 11 red cherry, RC; 15 pink large-fruited, PL; and 37 red large-fruited, RL) using hedonism scores and odor activity values. Taste compounds were detected using high-performance liquid chromatography. Volatiles were detected using gas chromatography–olfactometry–mass spectrometry. The flavor of tomato accessions can be evaluated using the DTOPSIS analysis method. According to the results of DTOPSIS analysis, 71 tomato accessions can be divided into 4 classes. Tomato accessions PL11, PC4, PC2, PC8, RL35, RC6, and RC10 had better flavor; accessions PC4, PC8, RC10, RL2, and RL35 had better tomato taste; and accessions PL11, PC2, and RC6 had better tomato odor. The concentrations of total soluble solids, fructose, glucose, and citric acid were shown to positively contribute to tomato taste. Tomato odor was mainly derived from 15 volatiles, namely, 1-hexanol, (*Z*)-3-hexen-1-ol, hexanal, (*E*)-2-hexenal, (*E*)-2-heptenal, (*E*)-2-octenal, (*E,E*)-2,4-decadienal, (*Z*)-3,7-dimethyl-2,6-octadieal, 2,6,6-timethyl-1-cyclohexene-1-carboxaldehyde, (2E)-3-(3-pentyl-2-oxiranyl)acrylaldehyde, 6-methyl-5-hepten-2-one, (*E*)-6,10-dimetyl-5,9-undecadien-2-one, methyl salicylate, 4-allyl-2-methoxyphenol, and 2-isobutylthiazole. Significant positive correlations (*P* < 0.05) were detected between the compound concentrations and flavor scores. The above-mentioned compounds can be used as parameters for the evaluation of flavor characteristics and as potential targets to improve the flavor quality of tomato varieties.

## Introduction

Tomato fruits are important dual-use (vegetable and fruit) products (Razifard et al., 2020). Because of their high nutritional value and various volatiles with delicious tastes and odors, tomato fruits are widely consumed worldwide (Zhu Y. et al., 2018). In 2018, tomato production reached 182.26 million tons all over the world (Food and Agriculture Organization of the United Nations [FAO], 2020). However, compared with heirloom tomatoes, modern commercial tomatoes have poor flavor, which can cause consumer dissatisfaction (Klee, 2010; Kanski et al., 2020). The flavor of tomato fruit mainly comes from soluble sugars, organic acids, amino acids, and volatile compounds. Compared with the wild or heirloom tomato varieties, modern tomato varieties have decreased many flavor compounds (fructose, glucose, citric acid, and at least 13 volatiles) throughout the process of domestication and improvement (Tomato Genome Consortium, 2012; Lin et al., 2014) because breeders serve growers, not consumers. Growers require tomato varieties with high yield, strong disease resistance, and a long shelf-life to ultimately ensure high returns (Giovannoni, 2018; Zhu G. et al., 2018; Razifard et al., 2020). A negative correlation has been observed between fruit weight and sugar concentration (Folta and Klee, 2016; Tieman et al., 2017). In order to obtain higher yield, breeders ignored the improvement of flavor quality. However, as living standards rise, consumers need not only sufficient food supplies but also nutritious, healthy, and delicious tomato fruits and are willing to pay more for them. Therefore, there is an increasing demand for the restoration of heirloom tomato flavors.

The challenge of improving modern tomato flavor has intrigued researchers. To achieve this, it is essential to understand the relationship between flavor compounds and sensory preferences (Franklin and Mitchell, 2019). As we know, the higher sugar and moderate acid make the sweetness and sourness ratio more suitable, nutritious, and delicious. The volatile profiles were primarily responsible for the differences in flavor across tomato varieties (Zhang et al., 2015). Through biochemical analysis and sensory evaluation, previous studies have found that taste compounds (soluble solids, fructose, glucose, and citric acid) and dozens of volatiles [6-methyl-5-hepten-2-one, (*E*)-6,10-dimetyl-5,9-undecadien-2-one, β-ionone, 2,5-dimethyl-4-hydroxy-3(2H)-furanone, (*E*)-2-pentenal, heptanal, (*E*)-2-heptenal, (*E,E*)-2,4-decadienal, benzaldehyde, phenylacetaldehyde, 1-pentanol, (*E*)-3-hexen-1-ol, 6-methyl-5-hepten-2-ol, 2-phenylethanol, 1-penten-3-one, 2-isobutylthiazole, 1-nitro-2-phenylethane, and 1-nitro-3-methylbutane] were significantly correlated with consumer preference and overall flavor intensity (Tieman et al., 2012, 2017; Tudor-Radu et al., 2016). Unfortunately, most of these flavor compounds have decreased in modern tomato varieties (Klee and Tieman, 2018). Thus, there is an urgent demand to increase the concentrations of sugar and preferable volatiles.

It is not supported by growers to sacrifice yield to increase sugar concentration of tomatoes (Goff and Klee, 2006). Volatiles may affect the flavor at very low concentrations, so they would be a candidate for flavor improvement without sacrificing yield. Volatile compounds are mainly derived from essential nutrients, such as fatty acids, carotenoids, and amino acids (Tieman et al., 2012), and can be responsible for tomato fruits having very different odor profiles. Volatiles can be affected by genotype, cultivation conditions, harvest-stage maturity, and postharvest treatment (Baldwin et al., 2015; Zhang et al., 2016; Zhao et al., 2019), which could change the level of precursor supply, gene expression, enzyme activity, and frequency of enzyme contact with substrates (Klee and Tieman, 2013). Although previous studies have conducted more comprehensive and deeper evaluations of tomato flavor compounds, the key tomato flavor compounds that are screened differ greatly in the published literature. To further clarify the flavor compounds of tomato and their effects on tomato flavor, we analyzed the taste compounds and volatiles, selected the key flavor factors through hedonism scores and odor activity values, and compared the flavor of 71 tomato accessions to supply reference data for the cultivation of tomato varieties with excellent flavor.

## Materials and Methods

### Tomato Materials

The 71 tomato accessions were inbred lines (Table 1), which were screened, segregated, and fixed by our lab—the Tomato Genetic Breeding and Quality Improvement Lab of Northwest A&F University. We used 8 pink cherry (PC1–PC8), 11 red cherry (RC1–RC11), 15 pink large-fruited (PL1–PL15), and 37 red large-fruited (RL1–RL8) tomato accessions. All of them are fresh market tomatoes rather than processing tomatoes. Seedling cultivation was conducted in a specialized seedling factory in January 2019. Tomato seedlings were then planted in a standardized research greenhouse in Yangling Zone (34°N, 108°E, 500 m altitude) of Shaanxi Province in March 2019. Tomatoes on the third inflorescence were picked when they reached the red ripe stage (i.e., mature tomato fruits with 90% surface coloring) (Shinozaki et al., 2018) from mid-June to early July 2019. Tomato samples were required to be in consistent size, have uniform coloring, and have no deformities, cleft fruit, or rot (Cheng et al., 2020).

**TABLE 1:** Concentrations of taste compounds of mature tomato fruits.

**Class**	**Accession**	**Evolution type**	**Soluble solids (%)**	**Fructose (mg 100 g**^–1^)	**Glucose (mg 100 g**^–1^)	**Citric acid (mg 100 g**^–1^)	**Malic acid (mg 100 g**^–1^)	**Sugar and acid ratio**
I	PL11	Large-fruited	6.03	1,290.00	838.50	251.33	144.17	5.38
	PC4	Cherry	8.17	1,801.20	1,023.15	451.92	266.03	3.93
	PC2	Cherry	7.23	2,148.80	1,220.60	267.21	157.29	7.94
	RL2	Large-fruited	4.77	1,376.67	840.00	332.82	235.60	3.90
	PC8	Cherry	6.80	2,391.07	1,358.22	243.89	143.57	9.68
	RL35	Large-fruited	5.23	1,465.17	894.00	178.67	126.48	7.73
	RC6	Cherry	7.13	1,420.83	955.83	264.18	57.35	7.39
	RC10	Cherry	9.63	2,145.00	1,443.00	461.72	100.23	6.38
	PL13	Large-fruited	4.97	1,120.00	728.00	278.40	159.70	4.22
	PC7	Cherry	7.07	1,716.93	975.28	347.91	204.80	4.87
II	RL33	Large-fruited	4.30	1,307.83	798.00	255.74	181.04	4.82
	RL19	Large-fruited	4.27	1,327.50	810.00	253.99	179.80	4.93
	PL7	Large-fruited	4.37	1,100.00	715.00	344.13	197.40	3.35
	PL9	Large-fruited	6.10	1,530.00	994.50	214.60	123.10	7.48
	RC8	Cherry	5.53	1,475.83	992.83	359.38	78.02	5.64
	PL4	Large-fruited	4.43	1,250.00	812.50	421.47	241.76	3.11
	PC3	Cherry	8.67	2,096.13	1,190.68	451.92	266.03	4.58
	RC9	Cherry	9.23	2,374.17	1,597.17	309.40	67.17	10.55
	PC1	Cherry	7.17	2,085.60	1,184.70	310.25	182.63	6.64
	RL20	Large-fruited	3.90	1,140.67	696.00	268.01	189.72	4.01
	RL12	Large-fruited	6.30	1,288.17	786.00	280.27	198.40	4.33
	RL36	Large-fruited	4.07	1,278.33	780.00	253.99	179.80	4.74
	PC6	Cherry	11.43	2,401.60	1,364.20	243.89	143.57	9.72
	RC1	Cherry	5.20	1,237.50	832.50	409.36	88.87	4.15
	RL28	Large-fruited	5.37	1,602.83	978.00	544.77	385.64	2.77
	RL26	Large-fruited	5.53	1,091.50	666.00	282.02	199.64	3.65
	RC7	Cherry	9.97	2,090.00	1,406.00	264.18	57.35	10.87
	PL6	Large-fruited	6.00	1,200.00	780.00	278.40	159.70	4.52
	RC5	Cherry	6.80	1,714.17	1,153.17	390.32	84.73	6.04
	RL8	Large-fruited	4.67	1,111.17	678.00	224.21	158.72	4.67
III	RL23	Large-fruited	6.10	1,091.50	666.00	210.20	148.80	4.90
	RC4	Cherry	7.03	1,815.00	1,221.00	514.08	111.60	4.85
	RL25	Large-fruited	6.97	1,593.00	972.00	259.25	183.52	5.79
	RL27	Large-fruited	5.93	1,396.33	852.00	120.87	85.56	10.89
	RL37	Large-fruited	6.40	1,229.17	750.00	194.44	137.64	5.96
	RL1	Large-fruited	5.03	1,337.33	816.00	201.44	142.60	6.26
	PL2	Large-fruited	5.47	1,270.00	825.50	280.33	160.81	4.75
	PL1	Large-fruited	6.37	1,560.00	1,014.00	320.93	184.09	5.10
	RL3	Large-fruited	4.53	1,337.33	816.00	283.77	200.88	4.44
	RL9	Large-fruited	5.63	1,307.83	798.00	218.96	155.00	5.63
	RC2	Cherry	8.03	1,925.00	1,295.00	254.66	55.28	10.39
	RL13	Large-fruited	6.23	1,317.67	804.00	252.24	178.56	4.92
	RL31	Large-fruited	6.80	1,219.33	744.00	178.67	126.48	6.43
	RL14	Large-fruited	4.43	1,071.83	654.00	197.94	140.12	5.11
	PL12	Large-fruited	6.33	1,650.00	1072.50	286.13	164.13	6.05
	PL3	Large-fruited	5.67	1,660.00	1,079.00	274.53	157.48	6.34
	PL15	Large-fruited	5.67	1,440.00	936.00	351.87	201.84	4.29
	RL16	Large-fruited	9.93	1,760.17	1,074.00	252.24	178.56	6.58
	RL6	Large-fruited	5.30	1,032.50	630.00	234.72	166.16	4.15
	RC11	Cherry	6.73	1,613.33	1,085.33	490.28	106.43	4.52
	RL21	Large-fruited	5.73	1,671.67	1,020.00	436.17	308.76	3.61
	PC5	Cherry	6.67	2,212.00	1,256.50	245.69	144.63	8.89
	RL10	Large-fruited	3.67	1,121.00	684.00	196.19	138.88	5.39
	PL5	Large-fruited	5.03	1,220.00	793.00	288.07	165.24	4.44
	PL14	Large-fruited	3.83	1,470.00	955.50	264.87	151.93	5.82
	RC3	Cherry	6.07	1,585.83	1066.83	323.68	70.27	6.73
	PL8	Large-fruited	5.37	1,050.00	682.50	255.20	146.39	4.31
	RL24	Large-fruited	6.10	1,091.50	666.00	199.69	141.36	5.15
IV	RL7	Large-fruited	4.27	1,366.83	834.00	199.69	141.36	6.45
	RL15	Large-fruited	6.07	1,268.50	774.00	183.93	130.20	6.50
	RL4	Large-fruited	5.53	1,121.00	684.00	294.28	208.32	3.59
	PL10	Large-fruited	6.67	1,850.00	1202.50	187.53	107.57	10.34
	RL17	Large-fruited	4.40	1,081.67	660.00	215.46	152.52	4.73
	RL22	Large-fruited	5.97	1,347.17	822.00	271.51	192.20	4.68
	RL32	Large-fruited	5.63	1,396.33	852.00	301.29	213.28	4.37
	RL5	Large-fruited	4.40	1,130.83	690.00	143.64	101.68	7.42
	RL29	Large-fruited	5.17	1,248.83	762.00	196.19	138.88	6.00
	RL11	Large-fruited	4.57	1,268.50	774.00	203.19	143.84	5.89
	RL30	Large-fruited	6.50	1,150.50	702.00	206.70	146.32	5.25
	RL18	Large-fruited	5.40	1,022.67	624.00	264.50	187.24	3.65
	RL34	Large-fruited	4.97	914.50	558.00	262.75	186.00	3.28

*PC, PL, RC, and RL indicated pink cherry tomato, pink large-fruited tomato, red cherry tomato, and red large-fruited tomato, respectively.*

### Experimental Methods

#### Soluble Solid

A small hole was made on the tomato, and a drop of tomato juice was squeezed out by hand and dropped on a PAL^–1^ digital refractometer (Atago Co., Ltd., Japan) to measure the concentration of soluble solid (Xu et al., 2018).

#### Soluble Sugars and Organic Acids

Fructose, glucose, citric acid, and malic acid concentrations were determined by HPLC (LC-2010A HT, Shimadzu Co., Ltd., Japan) with chromatographic column of C18 (Nucleodur 250 mm × 4.6 mm) (Acosta-Quezada et al., 2015; Zhao et al., 2016; Liu et al., 2017). The tomato fruits were crushed with a homogenizer (FJ200-SH, Specimen model factory, Shanghai, China). The non-polar metabolites of 0.001 kg sample were fractionated by 0.01 L chloroform. The polar metabolites were transferred into 0.05-L round bottom flask and dried under vacuum. Then, the sample was derivatized with methoxyamine hydrochloride and *N*-methyl-*N*-trimethylsilyltrifluoroacetamide (MSTFA) sequentially. After derivatization, the sample was filtrated twice by 0.22-μm microfiltration membrane for HPLC analysis. The citric acid and malic acid were also detected using an ultraviolet detector (VWD, Agilent Inc., United States). The mobile phase used to detect fructose and glucose was acetonitrile/water = 7:3 (v/v), and the mobile phase used to detect citric acid and malic acid was 0.2% metaphosphate. The flowrate was 1.67 × 10^–5^ L s^–1^. Column temperature was 35°C, and injection volume was 10^–5^ L.

#### Volatiles

Qualitative and quantitative analyses of volatile compounds were conducted using the headspace solid-phase microextraction (HS-SPME) gas chromatography–olfactometry–mass spectrometry (GC-O-MS) method (Farneti et al., 2017; Liu et al., 2019). Briefly, the samples were crushed with a homogenizer, and a 0.005-kg sample was added to 0.005 kg of anhydrous NaCl. The mix was vortexed to deactivate the tomato enzymes and filtered through a glass wool (Birtic et al., 2009). The supernatant was transferred to a 40-ml headspace bottle and added a magnetic rotor and 10^–5^ L of chromatographically pure 3.284 × 10^–5^ kg L^–1^ 3-nonanone (Meryer Chemical Technology Co., Ltd., Shanghai, China) were added. The volatile 3-nonanone was used as the internal standard because it is not found in tomato and is stable under normal temperature and pressure. The retention time (RT) of 3-nonanone appeared at 19.26 min. The RTs of volatiles in tomato appeared between 6.60 and 35.07 min. In the chromatogram, there are many volatiles peaks near to the peak of 3-nonanone, but the different peaks can be clearly distinguished. The recovery rate of 3-nonanone was as high as 98.423%. The headspace bottle containing the sample was placed on a magnetic stirrer (Troemner Inc., United States) for 2,400 s at 50°C. At the same time, the volatiles were extracted using an solid-phase microextraction (SPME) carboxen/polydimethylsiloxane (CAR/PDMS) fiber assembly (Product ID: 57318, 7.5 × 10^–5^ m; particle size, 0.01 m length) (Supelco Inc., United States). The fiber assembly was used in conjunction with the manual injection handle 57330U. As the temperatures increases, the fiber coating begins to lose its ability to adsorb analytes. Although the SPME fiber can be used at least 50 times, we replaced it after 45 uses. The volatiles were detected using the GC-MS instrument (ISQ & TRACE ISQ) (Thermo Fisher Scientific Inc., United States), which had a polar elastic quartz capillary chromatographic column HP-INNOWAX (0.25 mm i.d., 60 m length, 0.25 μm film thickness). In order to remove the residual solvents and volatiles from the filler and promote the uniform distribution and fixation of the liquid film on the filler surface, the SPME fiber and HP-INNOWAX column must be deactivated before extraction and detection of volatiles. The deactivation methods were as follows: the SPME fiber and HP-INNOWAX column were connected to the GC-MS instrument; the helium flowrate of carrier gas was 1.67 × 10^–4^ L s^–1^; the initial column temperature was 40°C, then increased to 230°C at a rate of 0.08°C s^–1^, and maintained for 7200 s.

The GC conditions used were as follows: inlet temperature of 230°C, over 99.999% helium carrier gas, no split injection, column flowrate of 1.67 × 10^–5^ L s^–1^, a split ratio of 20:1, and the splitless sample injection mode. The volatiles were desorbed for 150 s at 40°C. The column temperature was then increased to 110°C at a rate of 0.17°C s^–1^, increased to 230°C at a rate of 0.10°C s^–1^, and maintained for 600 s. The olfactory detector (OP275 Pro II, GL Sciences Inc., Japan) was connected at the outlet of the capillary column of GC, and the split ratio of MS and the olfactory detector was 1:1. The odor characteristics and intensities of detected volatiles were described and evaluated by five olfactory panelists at the outlet of the olfactometer (Majcher et al., 2020). The MS condition used as follows: ion source of EI, ion energy of 70 eV, full scan mode, scan range of 35–500 m z^–1^, ion source, and transmission line temperature of 230°C.

The RT of each normal alkane was measured after mixing the standard solutions of C4–C26 normal alkanes using GC-MS instrument. The retention index (RI) is known as the Kovats index, a parameter for the qualitative analysis of unknown compounds by gas chromatography (Matyushin et al., 2020). The RI of target volatile compound is calculated according to the RTs of the two normal alkanes adjacent to the target volatile compound (Matyushin et al., 2020). The RI of each volatile compound was calculated using the following equation (Chen et al., 2019):

RI=100Z+100[logt′R-(x)logt′R](z)/

(1) [logt′R-(z+1)logt′R](z)

where t′R is the RT, and Z and Z + 1 are the numbers of carbon atoms in the normal alkanes before and after the target volatiles (x) flow out, respectively. Note: t′R_(z)_ < t′R_(x)_ < t′R_(*z +* 1)_.

The qualitative analyses of volatiles were compared with the standard mass spectrum of the library (NIST2011, United States) and RI (Selli et al., 2014). Volatiles were assessed using mass spectrometry (Chen et al., 2020; Pu et al., 2020), and only those with both positive and negative matches > 800 (maximum of 1,000) were selected. The peak area normalization method was used to calculate the relative concentration of the various volatiles (Topi, 2020), as follows:

(2) m=n(S×nm)t/(S×tm)0

where m_*n*_ is the concentration (10^–9^ kg L^–1^) of the volatile compound (named “n”), S_*n*_ is the peak area of the volatile compound (named “n”), m_*t*_ is the internal standard (3-nonanone) concentration (10^–9^ kg L^–1^), S_*t*_ is the internal standard peak area, and m_0_ is the mass (kg) of the sample. Since tomato fruits were found to comprise up to 94.52% water (Petro-Turza, 1987), the mass of 1 L tomato homogenate is about 1 kg.

Volatile compounds are partitioned differently in the headspace and have different affinities for the polymer on the SPME fiber (Chambers and Koppel, 2013); therefore, the calibration curves of key volatiles were essential for quantitative analysis using the GC-MS method (Li et al., 2019), i.e., the measured volatile concentrations required correction according to their calibration curves. In this study, 60 chromatographically pure standards of volatile compounds (Meryer, China Chemical Technology Co., Ltd., Shanghai, China) were added to 0.005 kg water containing 10^–5^ L 3-nonanone (as the internal standard). The concentration gradient was formed by adding 0, 0.5 × 10^–5^, 1 × 10^–5^, 1.5 × 10^–5^, 2 × 10^–5^, 2.5 × 10^–5^, or 3 × 10^–5^ L standards, respectively. The compounds were measured under the same GC-MS conditions. Linear regression analysis was performed using the theoretical concentrations of volatile standards and the concentrations calculated by equation (1), as measured by GC-MS. The accurate concentration of each compound was calculated according to the corresponding calibration curve (Table 2) and Equation (2).

**TABLE 2:** Concentrations of volatiles in mature tomato fruits (10^–9^ kg L^–1^).

**Volatile compound**	**Abbreviation**	**Calibration curve^*a*^**	**Regression coefficient**	**Retention time (RT)**	**WAX retention index (RI)**	**Description^*b*^**	**Threshold concentration^*c*^**	**Concentration range**	**Average**	**Variation coefficient**	**OAV**
**Alcohols**											
3-Methyl-1-butanol	V1	*y* = 0.125x - 6E-05	0.997	11.43	1,210	Malty, solvent-like	250	17.3–1,328.78	108.26	0.34	<1
1-Pentan-3-ol	V2	*y* = 0.322x - 0.342	0.995	12.16	1,256	Green	400	20.01–1,036.58	109.26	0.34	<1
1-Nonanol	V3	*y* = 1.678x - 0.032	0.994	9.21	1,079	Roses, oranges, grease -like	50,000	20.62–556.15	142.28	2.24	<1
1-Hexanol	V4	*Y* = 2.987x - 2E-4	0.995	19.09	1,650	Resin, floral, green	500	208.8–24,033.28	3,109.08	1.47	6
(*Z*)-3-Hexen-1-ol*^d^	V5	*y* = 3.012x + 0.043	0.996	20.03	1,695	Lettuce-like	70	112.03–11,848.2	977.52	0.85	14
(*E*)-2-Hexen-1-ol	V6	*y* = 0.192x + 0.049	0.99	14.91	1,403	Fruity	3,900	24.74–1,303.4	239.72	1.19	<1
(*2Z*)-3,7-Dimethyl-2,6-octadien-1-ol	V7	*y* = 0.386x - 0.302	0.998	21.99	1,807	Rose-like	49,000	41.7–395.92	84.32	1.50	<1
2,4-Decadien-1-ol	V8	*y* = 0.186x + 0.004	0.995	25.27	2,042	Fatty, deep-fried	2,300	30.62–943.17	183.84	1.34	<1
(*E*)-2-Octen-1-ol	V9	*y* = 0.966x - 0.330	0.999	18.66	1,445	Mushroom, earthy	4,000	68.73–1,297.18	235.40	0.95	<1
6-Methyl-5-hepten-2-ol	V10	*y* = 0.128x + 0.051	0.993	22.03	1,810	Floral	2,000,000	1.15–124.13	15.85	1.04	<1
1-Octanol	V11	*y* = 0.326x - 0.467	0.996	17.8	1,582	Strong fatty citrus, rose-like	22,000	18.67–251.23	65.94	1.14	<1
2-Phenylethanol	V12	*y* = 0.142x - 0.009	0.996	31.74	2,373	Hyacinth, gardenia, nutty, fruity	140,000	44.43–1,227.32	255.21	1.56	<1
3,7-Dimethyl-6-octen-1-ol	V13	*y* = 0.023x + 0.008	0.991	26.64	2,148	Rose-like	46,500	50.86–256.63	146.80	0.78	<1
(*2E,6E,10E*)-3,7,11,15-Tetramethyl-2,6,10,14-hexadecatetraen-1-ol	V14	*y* = 0.765x - 0.232	0.991	24.46	1,982	Rose-like	50,000	2.3–16.52	7.15	1.48	<1
**Aldehydes**											
Hexanal*	V15	*y* = 2.433x - 0.561	0.997	11.48	1,213	Green, grassy	45	22.57–8,523.65	1,174.85	0.54	26
(*E*)-2-Hexenal*	V16	*y* = 1.39x + 0.004	0.992	14.96	1,406	Green apple-like, bitter	17	44.53–5,041.44	619.87	0.72	36
Non-anal	V17	*y* = 1.233x + 0.04	0.994	20.3	1,711	Citrus-like, soapy	28,000	15.89–397.18	124.18	1.20	<1
(*E*)-2-Octenal*	V18	*y* = 2.18x + 0.043	0.997	21.4	1,773	Mushroom, earthy	30	45.11–3,790.96	450.97	1.55	15
Decanal	V19	*y* = 4.268x + 5E - 3	0.994	23.08	1,887	Fatty, citrus-like, floral	30	25.46–1,343.38	165.74	0.69	6
(*E*)-2-Non-enal	V20	*y* = 1.043x + 0.031	0.992	17.54	1,570	Fatty, green	190	15.89–397.18	124.18	1.15	1
2,6,6-Timethyl-1-cyclohexene-1-carboxaldehyde*	V21	*y* = 0.026x - 8E-4	0.991	22.11	1,816	Mint, fruity	5	26.95–461.14	110.06	1.05	22
(*Z*)-3,7-Dimethyl-2,6-octadienal	V22	*y* = 1.897x + 0.006	0.994	20.28	1,709	Rose-like, citrus-like	30	26.7–1,360.92	190.66	0.69	6
(*E*)-3,7-Dimethyl-2,6-octadieal	V23	*y* = 4.548x + 0.072	0.992	21.11	1,757	Rose-like, citrus-like	1,100	2.4–239.64	65.65	0.75	<1
(*E,E*)-2,4-Decadienal*	V24	*y* = 2.041x - 0.033	0.99	29.28	2,278	Fatty, deep-fried	27	113.22–4,679.54	1,091.43	1.17	40
(*2E*)-3-(3-Pentyl-2-oxiranyl)acrylaldehyde	V25	*Y* = 0.038x - 0.309	0.998	25.84	2,086	Metallic	38	30.17–3,053.17	331.70	0.77	9
(*E*)-2-Heptenal*	V26	*Y* = 2.042x - 0.132	0.994	18.52	1,622	Green	13	20.92–1,219.46	195.73	0.78	15
5,9,13-Trimethyl-4,8,12-tetradecatrienal	V27	*y* = 0.712x + 0.034	0.997	30.15	2,312	–^e^	–	21.67–326	83.61	1.28	–
2-Undecenal	V28	*y* = 0.340x - 0.231	0.994	20.48	1,721	Fatty, floral, citrus-like	44,000	41.9–1,422.62	256.16	1.07	<1
(*E,E*)-2,4-Non-adienal	V29	*y* = 1.901x + 0.092	0.991	20.65	1,731	Fatty, green	62	39.15–676.24	200.43	0.86	3
(*2E,6E*)-3,7,11-Trimethyl-2,6,10-dodecatrienal	V30	*y* = 0.977x - 0.423	0.998	29.92	2,303	Soap-like, wax, violet, citrus-like	–	29.53–437.26	87.28	0.84	–
(*E*)-2-Decenal	V31	*y* = 1.347x - 0.036	0.996	19.53	1,672	Fatty	17,000	18.5–888.61	235.18	0.80	<1
**Ketones**											
1-Penten-3-one	V32	*y* = 2.096x + 0.003	0.992	9.77	1,113	Fruity, floral, green	940	17.14–221.37	71.28	0.89	<1
3-Octanone	V33	*y* = 1.129x - 0.332	0.995	12.44	1,272	Green, wax, vegetable-like	28,000	22.52–216.36	51.17	0.66	<1
1-Octen-3-one	V34	*Y* = 2.062x - 0.088	0.996	17.76	1,579	Mushroom-like	16	23.9–205.65	64.25	0.69	4
6,10-Dimethyl-2-undecanone,	V35	*Y* = 0.488x + 0.055	0.993	20.13	1,701	Wax, fruity, fatty	7,000	25.78–622.93	107.76	0.81	<1
(*E*)-6,10-Dimetyl-5,9-undecadien-2-one*	V36	*y* = 0.142x + 5E-4	0.994	23.16	1,893	Sweet, floral, ester-like	60	14.63–8,821.9	1,096.97	0.60	18
4-(2,6,6-Trimethyl-1-cyclohexen-1-yl)-3-buten-2-one	V37	*y* = 0.449x - 0.671	0.992	24.8	2,005	Floral, violet-like	3,500	4.05–321.43	67.08	1.27	<1
1-(2,6,6-Trimethyl-1-cyclohexen-1-yl)-2-buten-1-one*	V38	*y* = 2.190x + 0.038	0.993	26.51	2,138	Baked apple, grape-like	13	211.46–647.49	429.47	1.13	33
(*E,Z*)-6,10-Dimethyl-3,5,9-undecatrien-2-one	V39	*y* = 3.044x - 0.490	0.995	26.42	2,131	Balsamic, violet-like	800	3.62–131.1	34.99	0.94	<1
(*E,E*)-6,10,4-Trimethyl-5,9,13-pentadecatrien-2-one	V40	*Y* = 0.099x - 0.344	0.999	31.53	2,365	Fruity, floral	–	10.47–4,869.49	548.04	0.72	–
6-Methyl-5-hepten-2-one*	V41	*Y* = 1.432x - 0.043	0.99	18.82	1,637	Fruity, floral	50	29.84–11,923.39	1,376.41	0.77	28
6,10,14-Trimethyl-2-pentadecanone	V42	*y* = 1.376x - 0.233	0.999	27.56	2,210	Sweet, tea-like	15,000	1.99–42.93	8.80	1.15	<1
**Esters**											
2-Hydroxy-ethyl benzoate	V43	*y* = 0.877x - 9E-5	0.991	22.74	1,862	Herbs, sweet, spicy	900	45.1–6,378.2	1,225.83	1.34	1
Ethyl tetradecanate	V44	*Y* = 0.423x - 0.491	0.994	26.25	2,118	–	4,000,000	24.29–193.35	62.62	1.01	<1
Methyl hexadecanoate	V45	*Y* = 0.203x - 0.509	0.992	28.92	2,264	–	2,000,000	16.02–693.12	188.92	1.11	<1
Ethyl hexadecanoate	V46	*y* = 1.047x + 0,007	0.992	29.45	2,285	–	2,000,000	41–2,266.54	566.09	0.70	<1
Dimethyl phthalate	V47	*y* = 0.049x - 0.132	0.997	30.63	2,331	Light fragrance	–	22.97–305.44	63.94	0.82	–
Ethyl (*9Z,12Z*)-9,12-octadecadienoate	V48	*y* = 0.183x - 0.386	0.994	34.46	2,488	–	–	22.23–191.42	61.25	0.77	–
Hept-4-yl-isobutyl phthalate	V49	*y* = 0.345x + 0.057	0.998	35.07	2,516	Light fragrance	–	27.78–853.24	191.16	1.05	–
Ethyl octanoate	V50	*y* = 0.122x - 0.377	0.991	15.48	1,439	Pears litchi-like, sweet	13	28.94–180.99	71.64	0.65	6
Methyl salicylate*	V51	*y* = 0.071x + 0.003	0.994	29.71	2,295	Wintergreen, herbal	40	23.77–1,936.87	469.46	0.89	12
Ethyl acetate	V52	*y* = 4.823x - 0.061	0.999	6.61	942	Fruity	50,000	24.75–1,239.48	185.22	0.43	<1
Isopropyl palmitate	V53	*y* = 1.990x - 0.223	0.99	29.74	2,296	–	–	30.52–2,662	264.65	1.08	–
**Phenols**											
2,4-Bis(1,1-dimethylethyl)-phenol	V54	*y* = 0.055x - 0.367	0.995	30.39	2,321	Smoky, sweet	500,000	31.89–497.59	101.47	1.53	<1
4-Allyl-2-methoxyphenol*	V55	*y* = 0.032x + 0.001	0.997	28.52	2,249	Lilac, cinnamon, cantaloupe-like	20	34.31–1,752.73	330.04	2.51	17
2-Methoxyphenol	V56	*y* = 0.422x - 0.542	0.994	30.96	2,344	Smoky, sweet, woody, herbal	840	63.47–250.61	154.64	1.03	<1
**Other volatiles**											
Alanylglycine	V57	*y* = 0.058x - 0.399	0.998	3.84	487	Chicken-like	–	39.68–840.72	162.79	1.07	–
2-Pentyl-furan	V58	*y* = 1.099x + 0.383	0.99	14.93	1,404	Earthy, vegetable, malty, ham-like	6,000	42.09–1,157.35	301.08	0.60	<1
2-Isobutylthiazole*	V59	*y* = 1.049x + 0.097	0.996	20.74	1,736	Tomato vine, green	3.5	3.53–574.12	80.01	0.97	23
3-(4-methyl-3-pentenyl)-furan	V60	*y* = 0.328x - 0.539	0.994	15.32	1,429	Minty	–	21.95–201.22	63.22	0.73	–

*^*a*^In the calibration curve (*y* = kx + b), “y” represents the theoretical concentration of the labeled standard, and “x” represents the peak area ratio of a compound in the internal standard. ^*b*^Odor descriptions of volatiles come from the olfactory panelists and refer to the literature (Du et al., 2015; Kreissl and Schieberle, 2017; Zhu Y. et al., 2018). ^*c*^Odor thresholds of volatiles determined in water (10^–9^ kg L^–1^), reference to the literature (Kreissl and Schieberle, 2017; Zhu Y. et al., 2018). ^*d*^Volatiles that could be detected by an artificial olfactory system, as indicated by “^∗^” next to the compound name. ^*e*^“–” indicates the absence of data.*

#### Sensory Evaluation

Sensory evaluations of sweetness, sourness, characteristic flavor, and overall acceptability were conducted according to published methods (Tieman et al., 2012; Vallverdu-Queralt et al., 2013; Aisala et al., 2020), with slight modification. Briefly, different tomato accessions were numbered and cut into wedges (Cortina et al., 2018). After 7 days of training in the College of Food Science and Engineering, Northwest A&F University, the taste panels (25 male and 25 female, aged 18–60 years old) had mastered the taste evaluation methods (Tieman et al., 2012). Then, they conducted the sensory evaluations of 71 tomato accessions. To reduce the influence of visual preference on sensory evaluation, the sensory evaluators wore eye masks throughout the process. The maximum score was tentatively set at 8.00 points (Zhang K. et al., 2019). The taste panels scored the sweetness, sourness, characteristic flavor, and overall acceptability according to the taste intensity, e.g., the stronger the taste, the higher the score. After tasting each sample, the panels rinsed their mouths three times with purified water. To reduce taste fatigue, the panels conducted evaluations for 2,700-s periods (evaluate four to six tomato samples) and then took breaks of 900 s.

#### DTOPSIS Analysis

DTOPSIS analysis (Zhao et al., 2012) was used to evaluate the flavor of each tomato accession according to the following formula:

S=i+[Σ(R-i⁢jX)+j]2,1/2S=i-[Σ(R-i⁢jX)-j]2,1/2

(3) C=iS/i-(S+i-S)i+

where S_*i*_^+^ is the distance from the desirable flavor (X^+^_*j*_), S_*i*_^–^ is the distance from the undesirable flavor (X^–^_*j*_), and C_*i*_ is the closeness to the ideal fruit flavor.

### Statistical Analysis

All test data were recorded using WPS Office 2019. The standard deviation (SD) and coefficient of variation (CV) of the 71 tomato accessions were calculated using SPSS 22.0 (Zhang Y. et al., 2019). At least three biological replicates were performed for all flavor factors for each sample. The *Z*-score was used to standardize the data of tomato taste compounds, volatiles concentrations, and sensory evaluation scores (Ronningen et al., 2018). The significant differences in flavor among PC, PL, RC, and RL accessions were analyzed by a one-way ANOVA using SPSS 22.0. Pearson’s correlation of flavor compounds and sensory evaluation was analyzed using SPSS 22.0. The heatmap plots were prepared using the Heatmapper software^1^ (Sasha et al., 2016).

## Results

### Analysis of the Taste Compounds of Mature Tomato Fruits

**FIGURE 1:**
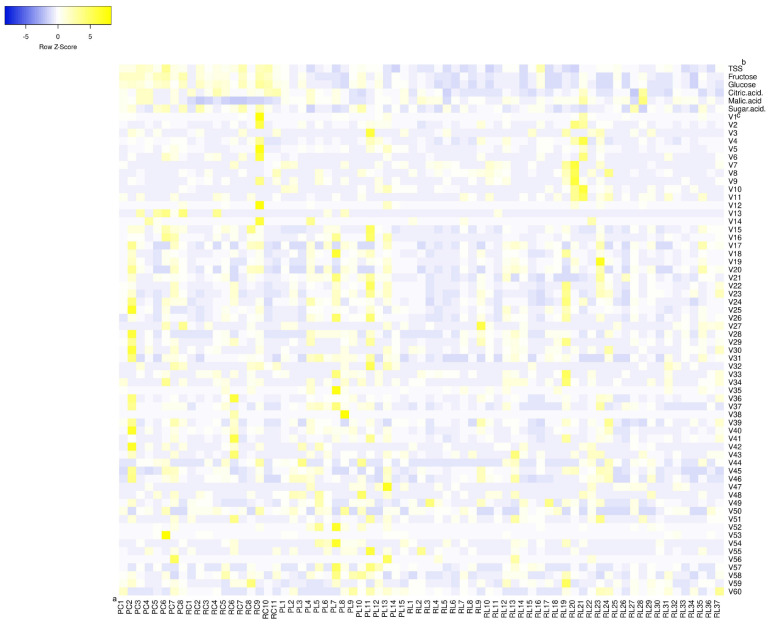
Heat map of concentrations of flavor compounds in mature tomato fruits. ^*a*^PC, PL, RC, and RL indicate pink cherry tomato, pink large-fruited tomato, red cherry tomato, and red large-fruited tomato, respectively. ^*b*^TSS represents the total soluble solids. ^*c*^V1–V60 represents the volatiles from top to bottom in Table 2, respectively.

The concentrations of taste compounds from the 71 tomato accessions are shown in Table 1, Supplementary Table S1, and Figure 1. The soluble solids, fructose, glucose, citric acid concentrations, and the sugar and acid ratio in cherry tomatoes were significantly higher than that in large-fruited tomatoes. RC had the lowest malic acid concentration. The soluble solids consisted mainly of fructose, glucose, citric acid, and malic acid. The concentration of fructose was higher (by 1.61-fold) than that of glucose, and the concentration of citric acid was higher (by 1.77-fold) than that of malic acid. Among the 71 tomato accessions, the concentration of soluble solids was higher in accessions PC6, RL16, RC7, RC10, and RC9 and ranged from 3.67 to 11.43%. Fructose concentrations ranged from 910 to 2,400 mg 100 g^–1^ and were highest in accessions PC6, PC8, RC9, PC5, and PC2. Glucose concentrations ranged from 560 to 1,600 mg 100 g^–1^ and were highest in accessions RC9, RC10, RC7, PC8, and PC6. Citric acid concentrations ranged from 120 to 540 mg 100 g^–1^ and were highest in accessions RL28, RC4, RC11, and RC10. Malic acid concentrations ranged from 60 to 390 mg 100 g^–1^ and were highest in accessions RL28, RC4, RC11, and PC4. The sugar and acid ratios ranged from 2.77 to 10.89, and accessions RL27, RC7, RC9, RC2, and PL10 had the highest ratios (above 10.00). Accessions RC9, PC6, RC7, and RC10 had the highest concentrations of taste compounds.

### Analysis of the Volatiles in Mature Tomato Fruits

A total of 60 volatiles were detected in this study (Table 2 and Figure 1). The concentrations of the total volatiles ranged from 3.67 to 53.37 × 10^–6^ kg L^–1^ (mean: 15.54 × 10^–6^ kg L^–1^). Accessions PL11, RC9, RC6, PC2, and RL21 had the highest total volatile concentrations. Classified by functional groups (Table 3 and Supplementary Table S2), there were 17 aldehydes, 14 alcohols, 11 ketones, 11 esters, 3 phenols, and 4 other volatiles detected, at concentrations of 5.51 × 10^–6^, 5.68 × 10^–6^, 3.73 × 10^–6^, 3.35 × 10^–6^, 4.85 × 10^–7^, and 6.07 × 10^–7^ kg L^–1^, respectively. Phenols were detected in only 28 of the 71 tomato accessions. Classified by metabolic precursors (Table 3 and Supplementary Table S3), there were 31 lipid-derived, 17 carotenoid-derived, 8 Phe-derived, and 5 Ile/Leu-derived volatiles detected, at concentrations of 1.03 × 10^–5^, 3.56 × 10^–6^, 1.43 × 10^–6^, and 5.59 × 10^–7^ kg L^–1^, respectively. Phe-derived volatiles were not detected in accession RC7. The common volatiles in the 71 accessions were 1-hexanol, (*Z*)-3-hexen-1-ol, hexanal, (*E*)-2-octenal, (*E*)-6,10-dimetyl-5,9-undecadien-2-one, (*E,E*)-6,10,4-trimethyl-5,9,13-pentadecatrien-2-one, and (*E,E*)-2,4-decadienal. The average concentration of each volatile in the 71 accessions was 2.59 × 10^–7^ kg L^–1^. The most abundant volatiles were 1-hexanol (mean: 3.11 × 10^–6^ kg L^–1^), 6-methyl-5-hepten-2-one, 2-hydroxy-ethyl benzoate, hexanal, (*E*)-6,10-dimetyl-5,9-undecadien-2-one, (*E,E*)-2,4-decadienal, (*Z*)-3-hexen-1-ol, and (*E*)-2-hexenal (mean: 6.20 × 10^–7^ kg L^–1^), which were mainly derived from lipids and carotenoids.

**TABLE 3:** Components and concentrations (10^–9^ kg L^–1^) of different groups of volatiles.

	**Component**	**Concentration**	**Range**	**Variation coefficient**	**Higher concentration accessions**
**Functional group**					
Alcohols	14	5,680.64	725.92–33,381.73	0.96	RC9, RL21, RL20, PL11, RL23
Aldehydes	17	5,507.68	710.70–22,161.77	0.74	PL11, PC2, PL7, RL19, PC6
Ketones	11	3,733.78	25.11–22,766.87	0.88	RC6, PC2, PL11, RL19, RL37
Esters	11	3,350.78	166.77–9,779.93	0.58	RL13, RC6, PC2, RL23, PL13
Phenols	3	484.69	0–1,752.73	0.58	PL11, RL2, PC7, PL13, PC4
Other volatiles	4	607.1	139.59–2,202.85	0.60	PL7, PL11, PL19, PL10, RC8
**Metabolic precursor**					
Lipid-derived	31	10,277.77	1,833.26–40,720.26	0.74	RC9, RL11, RL21, PC2, PC6
Carotenoid-derived	17	3,557.60	25.11–23,571.42	0.99	RC6, PC2, PL11, RL19, RL37
Phe-derived	8	1,427.94	0–8,860.50	1.23	RC6, PL13, RL23, PL11, PL13
Ile/Leu-derived	4	559.44	50.65–2,790.32	0.94	PL8, PL7, RC9, RC10, RL12

Only 1-hexanol in accessions RL21, RC9, RL20, PL11, (*Z*)-3-hexen-1-ol in accession RC9, and 6-methyl-5-hepten-2-one in accession RC6 were detected in concentrations higher than 10^–5^ kg L^–1^. The concentrations of volatiles, such as hexanal, (*E*)-2-hexenal, 2,6,6-timethyl-1-cyclohexene-1-carboxaldehyde, (*E,E*)-2,4-decadienal, (*E*)-2-decenal, 4-(2,6,6-trimethyl-1-cyclohexen-1-yl)-3-buten-2-one, (*E,E*)-6,10,4-trimethyl-5,9,13-pentadecatrien-2-one, ethyl (9Z,12Z)-9,12-octadecadienoate, and methyl hexadecanoate, were significantly higher in pink tomatoes than in red tomatoes. In addition, the concentrations of (2E,6E)-3,7,11-trimethyl-2,6,10-dodecatrienal, (*E,Z*)-6,10-dimethyl-3,5,9-undecatrien-2-one, and isopropyl palmitate were highest in PCs, and the concentrations of 1-nonanol, alanylglycine, and 2-pentyl-furan were the highest in pink PLs. Most volatiles were found in their lowest concentrations in RLs.

### Flavor Evaluation of Mature Tomato Fruits

#### Sensory Evaluation

**FIGURE 2:**
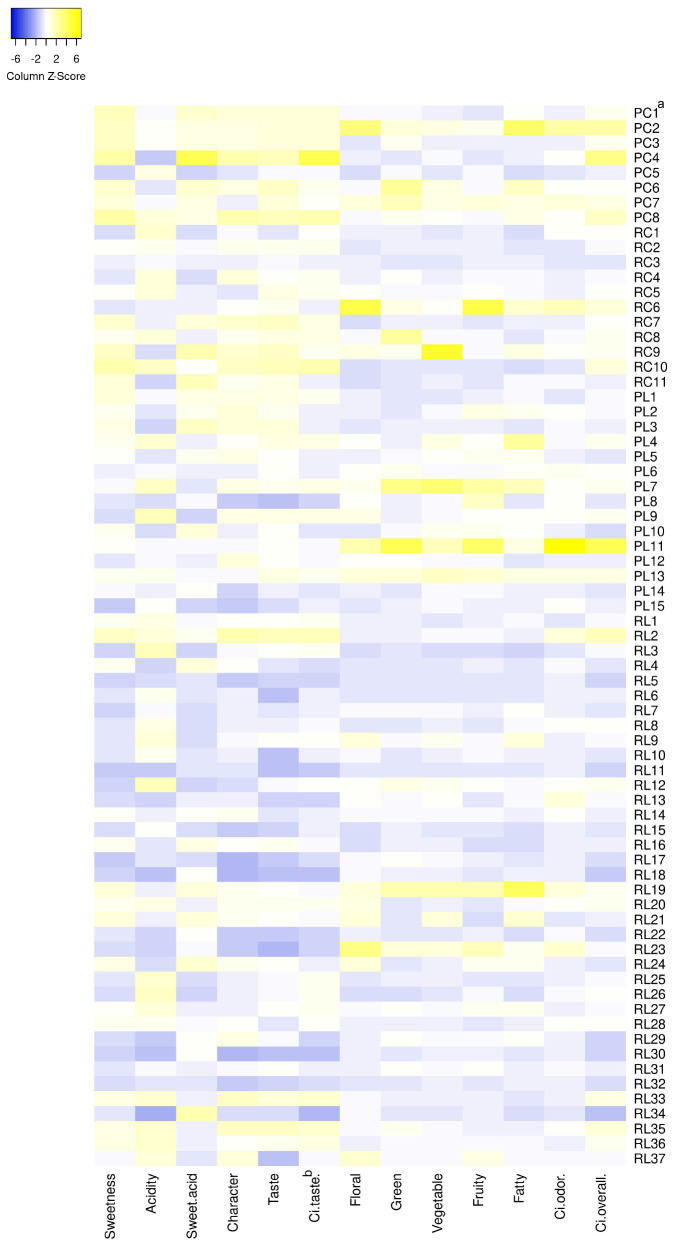
Heat map of the sensory evaluations of taste and odor characteristics of 71 tomato accessions. ^*a*^PC, PL, RC, and RL indicate pink cherry tomato, pink large-fruited tomato, red cherry tomato, and red large-fruited tomato, respectively. ^*b*^C_*i*_ values represent the scores of the DTOPSIS analysis.

The sensory evaluation scores are shown in Supplementary Table S4 and Figure 2. The taste factors included sweetness, sourness, the sweetness and sourness ratio, characteristic flavor, and overall acceptability. Sweetness, sweetness and sourness ratio, characteristic flavor, and overall acceptability were the strongest in PCs and the weakest in PLs. The sweetness scores ranged from 2.00 to 8.00 (mean ± CV: 4.33 ± 0.35) and were higher for accessions PC8, PC4, and RC10. The sourness scores ranged from 1.50 to 6.00 (4.13 ± 1.10) and were lower for accessions RL34, RL30, RL18, and PC4. The sweetness and sourness ratios ranged from 0.44 to 3.56 (1.16 ± 0.47) and were higher for accessions PC4, RC9, and RL34. The characteristic flavor scores ranged from 2.00 to 7.75 (5.00 ± 0.28) and were higher for accessions PC4, PC8, and RL2. The overall acceptability scores ranged from 1.75 to 8.00 (4.97 ± 0.33) and were higher for accessions PC4, PC8, RC10, and RL2. Accession PC4 had a high sweetness and sourness ratio and high characteristic flavor and overall acceptability scores. Accessions PC8, RC10, and RL2 had high characteristic flavor and overall acceptability scores.

Odor intensity can be quantified according to the ratio of the concentration to the olfactory threshold and is termed the “odor activity value” (OAV) (Buttery, 1993). In this study, the OAVs of 22 volatiles were >1 (Table 2), indicating that they had important impacts on flavor. Such volatiles are called odor-impact compounds (Baldwin et al., 2000). Of the 60 volatiles, 13 could be detected by an artificial olfactory system (as indicated by asterisk next to the compound names in Table 2). The volatiles that were found to contribute more to tomato flavor were (*E,E*)-2,4-decadienal, (*E*)-2-hexenal, 1-(2,6,6-trimethyl-1-cyclohexen-1-yl)-2-buten-1-one, 6-methyl-5-hepten-2-one, hexanal, 2-isobutylthiazole, 2,6,6-timethyl-1-cyclohexene-1-carboxaldehyde, (*E*)-6,10-dimetyl-5,9-undecadien-2-one, 4-allyl-2-methoxyphenol, (*E*)-2-heptenal, (*E*)-2-octenal, (*Z*)-3-hexen-1-ol, and methyl salicylate. The highest OAV was reported for accession PL11 (OAV = 1,196), and the lowest OAV was reported for accession RL26 (OAV = 79). The components of the odor-impact compounds ranged from 8 (accession RL26) to 21 (accession PL8).

According to the characteristic description, the volatile odors could be divided into green, floral, fruity, vegetable, fatty, and irritant odors. The intensities of green and fatty odors were the strongest in PCs, and the weakest in PLs. The OAVs of green odor ranged from 11 to 574 (mean ± CV: 98 ± 1.35) and were the highest for accessions PL11, PL7, and PC6. The OAVs of floral odor ranged from 5 to 175 (36 ± 0.82) and were the highest for accessions RC6, PC2, and RL23. The OAVs of fruity odor ranged from 4 to 258 (51 ± 0.88) and were the highest for accessions RC6, PL11, and PL7. Accessions RL19 and PL7 had stronger green, vegetable, and fruity odors. The OAVs of fatty odor ranged from 4 to 184 (42 ± 0.79) and were the highest for accessions RL19, PC2, and PL4. The OAVs of vegetable odor ranged from 6 to 194 (31 ± 0.96) and were the highest for accessions RC9, PL7, and RL19. The OAVs of irritant odor were lower than 153 (22 ± 1.14). No irritation odors were detected in accessions PC8, PL3, RL26, and RL31. “Irritant odors” represent the disliked odors and were mainly attributed to 2-hydroxy-ethyl benzoate (herbs, sweet, spicy), methyl salicylate (wintergreen, herbal), 2,4-bis(1,1-dimethylethyl)-phenol (smoky, sweet), 4-allyl-2-methoxyphenol (lilac, cinnamon, cantaloupe-like), and 2-methoxyphenol (smoky, sweet, woody, herbal).

#### DTOPSIS Analysis

The DTOPSIS analysis method was used to evaluate the flavors of the different tomato accessions (Supplementary Table S4 and Figure 2). Among all the sensory factors, the sourness and irritant odor were negative indicators of flavor, while the others were positive factors. To rank accessions according to taste and odor factors, each sensory factor was simplified as a unique score (Cortina et al., 2018). The scores and rankings of the tomato flavor evaluation [i.e., results from Equation (3)] are shown in Supplementary Table S1. The taste evaluation [**C_*i*_**_(taste)_], odor evaluation [**C_*i*_**_(__*odor*__)_], and comprehensive flavor evaluation [**C**_*i(overall)*_] values were -2.05–4.45, -0.64–6.85, and -2.69–6.70, respectively. The 71 tomato accessions can be divided into four classes according to [**C**_*i(overall)*_] from high to low, contains 10, 20, 28, and 13 tomato accessions, respectively (Table 1 and Supplementary Table S4). Of the 71 tomato accessions assessed, the flavor scores were higher in accessions PL11, PC4, PC2, PC8, RL35, RC6, and RC10; among these, accessions PC4, PC8, RC10, RL2, and RL35 had better tomato taste, and accessions PL11, PC2, and RC6 had better tomato odor. The intensities of the green and fatty odors were the strongest in PCs and the weakest in PLs. The [**C_*i*_**_(taste)_] and [**C**_*i(overall)*_] values were significantly higher in PCs than in the other types of tomatoes.

### Correlation Analyses of Key Flavor Factors in Mature Tomato Fruits

The results of the Pearson’s correlation analyses between compound concentrations and flavor intensities are shown in Table 4. The [**C**_*i(overall)*_] values were significantly positively (*P* < 0.05) correlated with the concentrations of glucose, citric acid, (*E*)-6,10-dimetyl-5,9-undecadien-2-one, and (*E,Z*)-6,10-dimethyl-3,5,9-undecatrien-2-one. Furthermore, the [**C**_*i(overall)*_] values were very significantly positively (*P* < 0.01) correlated with fructose, 1-nonanol, hexanal, (*E*)-2-hexenal, (*E*)-2-octenal, (*E*)-2-heptenal, 2-undecenal, (*E,E*)-2,4-nonadienal, (*E*)-2-decenal, 1-penten-3-one, 2,6,6-timethyl-1-cyclohexene-1-carboxaldehyde, (*Z*)-3,7-dimethyl-2,6-octadienal, (*E*)-3,7-dimethyl-2,6-octadieal, (*E,E*)-2,4-decadienal, (2E)-3-(3-pentyl-2-oxiranyl) acrylaldehyde, 4-(2,6,6-trimethyl-1-cyclohexen-1-yl)-3-buten-2-one, (*E,E*)-6,10,4-trimethyl-5,9,13-pentadecatrien-2-one, 6-methyl-5-hepten-2-one, methyl salicylate, and 4-allyl-2-ethoxyphenol, i.e., mainly aldehydes. The [**C**_*i(overall)*_] values were also very significantly positively correlated with sweetness, sourness, characteristic flavor, overall acceptability, floral, fruity, fatty, green, and irritation odors. The soluble solids, fructose, glucose, and citric acid were significantly positively correlated with the [**C_*i*_**_(taste)_] values, sweetness, sweetness and sourness ratio, characteristic flavor, and overall acceptability. Many volatiles were significantly positively correlated with the [**C_*i*_**_(__*odor*__)_] values, fatty, green, floral and fruity, vegetable, and irritant odors. According to metabolic precursors, the carotenoid-derived volatiles were significantly positively correlated with the floral and fruity odors; the lipid-derived volatiles were significantly positively correlated with the green, floral, and fruity odors; the Ile/Leu-derived volatiles were significantly positively correlated with the green and vegetable odors; and the Phe-derived volatiles were significantly positively correlated with the irritant odor.

**TABLE 4:** Correlations between flavor compounds and sensory evaluations in mature tomato fruits.

	**C**${}_{i\,\left(o\,v\,e\,r\,a\,l\,l\right)}^{a}$	**C_*i*_**_(taste)_	**C_*i*_**_(__*odor*__)_	**Sweetness**	**Sweetness/sourness**	**Characteristic flavor**	**overall acceptability**	**Floral**	**Green**	**Vegetable**	**Fruity**	**Fatty**	**Irritation**
Soluble solids	0.219	0.295*	0.023	0.437**^b^	0.406**	0.301*^c^	0.445**	0.016	0.136	0.121	−0.053	0.004	0.021
Fructose	0.313**	0.495**	−0.040	0.593**	0.365**	0.491**	0.643**	−0.045	0.119	0.170	−0.152	0.159	−0.067
Glucose	0.283*	0.457**	−0.045	0.556**	0.332**	0.489**	0.649**	−0.047	0.098	0.204	−0.118	0.120	−0.073
Citric acid	0.248*	0.377**	−0.016	0.363**	0.248*	0.290*	0.339**	−0.098	−0.024	0.052	−0.113	0.036	0.011
Sugar/acid	0.059	0.135	−0.050	0.214	0.096	0.216	0.338**	0.038	0.108	0.132	0.011	0.083	−0.085
3-Methyl-1-butanol	0.050	0.081	−0.009	0.229	0.273*	0.169	0.194	0.130	0.028	0.687**	−0.046	0.125	0.012
1-Nonanol	0.393**	−0.128	0.700**	−0.002	0.042	−0.128	−0.073	0.294*	0.498**	0.187	0.442**	0.244*	0.573**
1-Hexanol	0.226	0.075	0.254*	0.173	0.152	0.112	0.125	0.416**	0.210	0.512**	0.137	0.225	0.214
(***Z*)-3-Hexen-1-ol**	0.193	0.093	0.187	0.260*	0.310**	0.140	0.218	0.259*	0.228	0.790**	0.132	0.203	0.179
(***E***)-2-Hexen-1-ol	0.035	0.077	−0.026	0.284*	0.311**	0.201	0.255*	0.160	0.112	0.639**	−0.052	0.238*	−0.033
2-Phenylethanol	0.096	0.104	0.035	0.232	0.243*	0.157	0.209	0.151	0.070	0.682**	0.010	0.114	0.049
Hexanal	0.551**	0.153	0.648**	0.155	−0.020	0.109	0.228	0.396**	0.877**	0.379**	0.508**	0.308**	0.516**
(***E*)-2-Hexenal**	0.479**	0.065	0.631**	0.129	0.044	0.086	0.209	0.376**	0.893**	0.577**	0.619**	0.348**	0.496**
(***E*)-2-Octenal**	0.307**	0.087	0.359**	0.087	−0.010	0.125	0.133	0.426**	0.682**	0.697**	0.596**	0.699**	0.302*
Decanal	0.155	−0.057	0.282*	0.004	−0.008	−0.127	−0.096	0.521**	0.245*	0.226	0.217	0.210	0.329**
2,6,6-Timethyl-1-cyclohexene-1-carboxaldehyde	0.361**	0.097	0.427**	0.022	−0.103	0.030	0.109	0.322**	0.587**	0.440**	0.506**	0.312**	0.322**
(***Z*)-3,7-Dimethyl-2,6-octadienal**	0.495**	−0.045	0.765**	0.062	0.018	0.009	0.011	0.648**	0.724**	0.493**	0.839**	0.576**	0.655**
(***E***)-3,7-Dimethyl-2,6-octadieal	0.380**	−0.064	0.615**	0.048	0.023	0.019	−0.035	0.723**	0.628**	0.481**	0.788**	0.621**	0.563**
(***E,E*)-2,4-Decadienal**	0.360**	0.177	0.346**	0.350**	0.195	0.247*	0.302*	0.582**	0.475**	0.591**	0.472**	0.998**	0.356**
(***2E*)-3-(3-Pentyl-2-oxiranyl)acrylaldehyde**	0.405**	0.154	0.434**	0.208	0.119	0.101	0.154	0.481**	0.385**	0.381**	0.263*	0.655**	0.494**
(***E*)-2-Heptenal**	0.452**	0.056	0.600**	0.032	−0.065	0.063	0.088	0.481**	0.810**	0.674**	0.692**	0.601**	0.487**
2-Undecenal	0.336**	0.114	0.374**	0.080	−0.012	0.093	0.083	0.427**	0.280*	0.295*	0.177	0.573**	0.435**
(***E,E*)-2,4-Nonadienal**	0.483**	0.098	0.603**	0.111	0.017	0.112	0.123	0.605**	0.460**	0.378**	0.546**	0.731**	0.590**
(***2E,6E***)-3,7,11-Trimethyl-2,6,10-dodecatrienal	0.217	0.102	0.214	0.212	0.184	0.085	0.029	0.400**	0.040	0.142	0.067	0.345**	0.349**
(***E***)-2-Decenal	0.483**	0.180	0.521**	0.251*	0.101	0.214	0.257*	0.420**	0.494**	0.381**	0.401**	0.596**	0.530**
1-Penten-3-one	0.433**	0.035	0.595**	0.188	0.134	−0.016	0.147	0.184	0.533**	0.359**	0.382**	0.059	0.437**
1-Octen-3-one	0.022	0.014	0.017	0.150	0.089	0.094	0.095	0.030	0.309**	0.287*	0.132	0.390**	−0.019
(***E*)-6,10-Dimetyl-5,9-undecadien-2-one**	0.297*	−0.002	0.432**	0.014	−0.014	0.047	0.023	0.889**	0.239*	0.197	0.658**	0.501**	0.552**
4-(2,6,6-Trimethyl-1-cyclohexen-1-yl)-3-buten-2-one	0.340**	0.128	0.366**	0.140	0.039	0.069	0.190	0.408**	0.493**	0.544**	0.464**	0.623**	0.356**
1-(2,6,6-Trimethyl-1-cyclohexen-1-yl)-2-buten-1-one	−0.035	−0.143	0.093	−0.103	−0.032	−0.161	−0.176	0.184	−0.031	0.005	0.337**	−0.028	0.149
(***E,Z***)-6,10-Dimethyl-3,5,9-undecatrien-2-one	0.248*	0.038	0.320**	0.150	0.109	0.131	0.089	0.588**	0.254*	0.213	0.374**	0.588**	0.361**
(***E,E***)-6,10,4-Trimethyl-5,9,13-pentadecatrien-2-one	0.372**	0.143	0.398**	0.190	0.103	0.122	0.158	0.688**	0.223	0.249*	0.365**	0.628**	0.519**
6-Methyl-5-hepten-2-one	0.385**	−0.078	0.637**	−0.006	−0.021	0.019	0.017	0.745**	0.544**	0.342**	0.914**	0.461**	0.595**
6,10,14-Trimethyl-2-pentadecanone	0.225	0.097	0.229	0.254*	0.241*	0.166	0.184	0.393**	0.088	0.142	0.118	0.457**	0.307**
Methyl salicylate	0.345**	−0.182	0.683**	−0.132	−0.105	−0.166	−0.231	0.642**	0.316**	0.124	0.569**	0.156	0.763**
4-Allyl-2-methoxyphenol	0.634**	0.140	0.781**	0.113	0.091	0.107	0.154	0.269*	0.372**	0.116	0.447**	0.032	0.764**
2-Pentyl-furan	0.191	0.087	0.191	0.098	0.007	0.181	0.220	0.186	0.453**	0.443**	0.368**	0.491**	0.125
2-Isobutylthiazole	0.143	0.093	0.113	0.118	−0.011	0.081	0.138	0.238*	0.529**	0.262*	0.291*	0.457**	0.094
3-(4-methyl-3-pentenyl)-furan	0.085	−0.148	0.272*	−0.077	−0.069	−0.047	−0.205	0.239*	0.176	0.018	0.345**	0.050	0.210

^*a*^C_*i*_ values represent the scores of the DTOPSIS analysis. ^b^** means a very significant correlation (*P* < 0.01). ^c^* means significant correlation (*P* < 0.05).

There was no significant correlation between taste compounds and odor characteristics, but some volatiles showed significant positive correlations with the taste evaluations. The overall acceptability was significantly positively correlated with (*E*)-2-hexen-1-ol and (*E*)-2-decenal. The characteristic flavor was significantly positively correlated with (*E,E*)-2,4-decadienal. The sweetness and sourness ratio were significantly positively correlated with 3-methyl-1-butanol, 2-phenylethanol, and ethyl tetradecanoate. Sweetness was significantly positively correlated with (*E,E*)-2,4-decadienal.

## Discussion

Flavor involves both taste and odor and is perceived by the binding of taste compounds and volatiles to sensory receptors. The human taste system can detect five to seven tastes (sweet, sour, salty, bitter, hemp, umami, and koukumi) and the olfactory system can detect thousands of odors. Sweetness and sourness were the basis of tomato flavor (Baldwin et al., 2008; D’angelo et al., 2018). In a previous study, tomato fruits were found to comprise up to 94.52% water, in addition to fructose, glucose, citric acid, and malic acid concentrations accounting for 25, 22, 9, and 4% of the dry weight of tomato, respectively (Petro-Turza, 1987). In addition, this previous study reported the concentrations of fructose, glucose, citric acid, and malic acid in fresh tomato fruits to be 1,370, 1,205.6, 493.2, and 219.2 mg 100 g^–1^, respectively. Another previous study reported consistent values, i.e., fructose and glucose concentrations of 1,370 and 1,250 mg 100 g^–1^, respectively (United States Department of Agriculture [USDA], 2011). The concentrations of fructose, glucose, citric acid, and malic acid in tomato genotype “Fla.8153” were 1.20, 1.11, 0.31, and 0.04%, while they were 0.99, 0.96, 0.34, and 0.07% in tomato genotype “Florida 47,” respectively. In the present study, we reported fructose, glucose, citric acid, and malic acid concentrations of 1,464.36, 911.72, 281.50, and 158.81 mg 100 g^–1^, respectively (Baldwin et al., 2015). Compared to the wild-type *Solanum pimpinellifolium* (Çolaka et al., 2020), tomato fruits in this study have lower glucose and fructose (decreased by 75%) and higher malic acid and citric acid (by 40-fold). The decreased sugar and increased malic acid concentrations, and resulting higher sourness, are prominent issues in modern tomato fruits.

The sweetness of tomato fruits are mainly attributed to their fructose and glucose concentrations. The (*E*)-6,10-dimetyl-5,9-undecadien-2-one, ethyl octanoate, and 2-hydroxy-ethyl benzoate volatiles were perceived to be sweet. Baldwin et al. (1998) also found that sweetness was closely correlated with glucose, fructose, (*E*)-6,10-dimetyl-5,9-undecadien-2-one, hexanal, (*Z*)-3-hexenal, (*E*)-2-hexenal, and (*Z*)-3-hexenol. The sweetness and the sweetness and sourness ratio were significantly higher in PCs than in the other three types tomatoes, which resulted in the overall acceptability and tomato-like flavor of PCs being perceived as more delicious. Decrease in sweetness is a prominent issue in modern tomato fruits, which can be improved by increasing the concentrations of sugars and volatiles with sweetness perception. However, sugar concentrations are reportedly negatively correlated with fruit weight (Folta and Klee, 2016). The most promising way to improve the sweetness of tomatoes is via the promotion of certain volatiles. Such investigations have great potential and are worth undertaking because (1) the concentrations of volatiles in tomato fruits are very low and can be increased greatly without affecting the yield or fruit size; there is an urgent requirement for the concentrations of consumer-preferred volatiles to be increased (Tieman et al., 2017) to improve the flavor intensity and variation in modern tomatoes.

The development of GC-O-MS has led to the identification of thousands of volatiles (El Hadi et al., 2013; Pott et al., 2019), and numerous odor characteristics are distinguishable by the developed human sense of olfactory (Pavagadhi and Swarup, 2020). Only a few dozen volatiles contribute substantially to flavor and only when their concentrations exceed the olfactory threshold (Czerny et al., 2008). Most volatiles can only be used as background odors (Tieman et al., 2012). This study identified 22 odor-impact compounds, 12 of which were consistent with those reported in the literature, e.g., hexanal, (*E*)-2-hexenal, (*E*)-2-heptenal, 2,6,6-timethyl-1-cyclohexene-1-carboxaldehyde, (*Z*)-3,7-dimethyl-2,6-octadienal, 1-hexanol, (*Z*)-3-hexen-1-ol, (*E*)-6,10-dimetyl-5,9-undecadien-2-one, 1-(2,6,6-Trimethyl-1-cyclohexen-1-yl)-2-buten-1-one, 6-methyl-5-hepten-2-one, methyl salicylate, and 2-isobutylthiazole (Tieman et al., 2012; Vallverdu-Queralt et al., 2013; Du et al., 2015). (*E,E*)-2,4-decadienal, 1-octen-3-one, (*E*)-2-nonenal, (2E)-3-(3-pentyl-2-oxiranyl)acrylaldehyde, and 4-allyl-2-methoxyphenol were found to be the important volatiles of tomato (Kreissl and Schieberle, 2017; Tieman et al., 2017). In the present study, decanal, (*E*)-2-octenal, (*E,E*)-2,4-nonadienal, ethyl octanoate, and 2-hydroxy-ethyl benzoate were shown to be important contributing volatiles to the flavor.

The OAVs of odor-impact compounds ranged from high to low for (*E,E*)-2,4-decadienal, (*E*)-2-hexenal, 1-(2,6,6- Trimethyl-1-cyclohexen-1-yl)-2-buten-1-one, 6-methyl-5-hep ten-2-one, hexanal, 2-isobutylthiazole, 2,6,6-timethyl-1-cyclohexene-1-carboxaldehyde, and (*E*)-6,10-dimetyl-5,9-undecadien-2-one. Contrarily, the OAVs in a previous study on Italian tomatoes revealed (*Z*)-3-hexenal, (2E)-3-(3-pentyl-2-oxiranyl)acrylaldehyde, hexanal, wine lactone, (*E*)-β-damascenone, 1-penten-3-one, 1-octen-3-one, and (*E,E*)-2,4-decadienal to be the main odor contributors (Kreissl and Schieberle, 2017). The profiles of volatiles in tomato fruits appear to vary greatly among different genotypes and regions. Pearson’s correlation was used to indicate the contribution of chemicals to flavor. Fructose, glucose, citric acid, and 21 volatiles showed significant positive correlations (*P* < 0.05) with the [**C**_*i(overall)*_] values. To our knowledge, the positive flavor characteristics are represented by the characteristic tomato taste, overall acceptability, sweetness, and floral, fruity, green, and vegetable odors, which are mainly derived from fructose, glucose, lipid-derive volatiles [hexanal, (*E*)-2-hexenal, (*E*)-2-heptenal, (*E,E*)-2,4-decadienal, 1-hexanol, (*Z*)-3-hexenol], carotenoid-derive volatiles [6-methyl-5-hepten-2-one, (*E*)-6,10-dimetyl-5,9-undecadien-2-one] (Vogel et al., 2010), 2-phenylethanol, and 2-isobutylthiazole (Carbonell-Barrachina et al., 2005; Bartoshuk and Klee, 2013; Socaci et al., 2014; Klee and Tieman, 2018). The factors disliked by consumers were high sourness, phenolic odors, and pungent odors, which were mainly associated with the pH (Cohen et al., 2014; Tudor-Radu et al., 2016), esters (butyl acetate), Ile/Leu-derived volatiles (3-methyl-1-butanol), and Phe-derived volatiles (methyl salicylate and 2-methoxyphenol) (Piombino et al., 2013; Vallverdu-Queralt et al., 2013). In the present study, the volatiles with preferred odors and lower threshold concentrations were hexanal, (*E*)-2-hexenal, (*E*)-2-octenal, 2,6,6-timethyl-1-cyclohexene-1-carboxaldehyde, (*Z*)-3,7-dimethyl-2,6-octadienal, (*E,E*)-2,4-decadienal, (*E*)-2-heptenal, (*E,E*)-2,4-nonadienal, 1-penten-3-one, (*E*)-6,10-dimetyl-5,9-undecadien-2-one, 4-(2,6,6- trimethyl-1-cyclohexen-1-yl)-3-buten-2-one, (*E,Z*)-6,10-dimethyl-3,5,9-undecatrien-2-one, (*E,E*)-6,10,4-trimethyl-5,9, 13-pentadecatrien-2-one, and 6-methyl-5-hepten-2-one. Their concentrations should be increased. Only malic acid, (2E)-3-(3-pentyl-2-oxiranyl)acrylaldehyde (metallic), 2-hydroxy-ethyl benzoate (herbs, sweet, spicy), methyl salicylate (wintergreen, herbal), and 2-methoxyphenol (smoky, sweet, woody, herbal) were disliked by the “consumers” (i.e., represented by the evaluation panels) and should, therefore, be reduced in tomato fruits. Baldwin et al. (1998) found that (*E*)-6,10-dimetyl-5,9-undecadien-2-one and 6-methyl-5-hepten-2-one had preferable odors in tomato fruits. Tieman et al. (2017) identified 33 chemicals linked to consumer preferences and 37 linked to the flavor intensity. Unexpectedly, several characteristic volatiles were not significantly correlated with consumer liking, such as (*E*)-2-hexenal, 1-penten-3-ol, 3-methyl-1-butanol, hexanol, and methyl salicylate (Klee and Tieman, 2018). The present study showed that both (*E*)-2-hexenal (0.479^∗∗^) and methyl salicylate (0.345^∗∗^) were very significantly positively correlated with the [**C**_*i(overall)*_] values.

The sensory evaluation found a low correlation (only 0.06) between the taste score and odor intensity. The concentrations of taste compounds were not significantly correlated with odor intensity, and only several volatiles were significantly correlated with the taste score. These findings were consistent with those of a previous study, in which consumers largely considered sweet and sour to be important contributions to flavor (Andersen et al., 2019). Sugar and acid are considered to be the basic compounds for fruit flavor formation (Baldwin et al., 2008; Bastias et al., 2011), while the volatiles form the characteristic flavor compounds of different fruits (Baldwin et al., 2008; Du et al., 2015). Therefore, it is not scientifically valid to judge tomato flavor on taste alone; instead, taste and odor should both be assessed to comprehensively evaluate the flavor (Pavagadhi and Swarup, 2020). The results of the DTOPSIS evaluation in the present study indicated that tomato accessions with a preferred (i.e., “better”) flavor either scored high in the taste evaluation or had a strong odor.

The enhancement of consumer-preferred tomato fruits volatiles should be expected. From the perspective of metabolism, these preferred volatiles mainly come from fatty acids, carotenoids, and amino acids (Rambla et al., 2014; Wang et al., 2015; Lee et al., 2019), and the key enzymes involved in their production are lipase, lipoxygenase, hydroperoxidase lyase (Chen et al., 2004; Garbowicz et al., 2018), carotenoid cleavage dioxygenase (Simkin et al., 2004; Ilg et al., 2014), phenylalanine ammonia-lyase, phenylacetaldehyde reductases, *o*-methyltransferases (Tieman et al., 2007; Tieman et al., 2010; Gapper et al., 2013), and branched-chain amino acid aminotransferase (Maloney et al., 2010). Many quantitative trait loci have been identified (Lin et al., 2014; Liu et al., 2016; Bauchet et al., 2017; Kimbara et al., 2018; Ferrao et al., 2020), and the absence of relevant alleles has been noted in modern tomatoes (Zhu G. et al., 2018; Liu et al., 2020). The reintroduction of certain alleles in modern tomatoes may be a feasible option to improve their flavor (Rigano et al., 2014; Zhao et al., 2019). For example, the rare allele of the lipoxygenase C promoter has been shown to increase the concentrations of carotenoid-derived volatiles (Gao et al., 2019).

In the present study, only the free volatiles were analyzed, but the glycoside-bonded volatiles also account for a certain proportion of volatiles in tomato fruits. Glycoside-bonded volatiles serve as reserve odors and can be hydrolyzed by enzymes and acids to free volatiles (Schwab et al., 2015). Although the taste and odors of 71 tomato accessions were evaluated based on taste compounds and volatiles, the complexity of flavor and individual differences in the sensory evaluations complicates the process of improving tomato fruits flavor. Additionally, flavor factors can interact with each other; in which case both the concentration and proportion of the compounds affect the production of “good” flavors. Comprehensive investigations are still needed to effectively improve the flavor of tomato fruits. Driven by the motivation to breed delicious tomatoes and using multidisciplinary approaches that involve biological evolution, multiple omics, flavor chemistry, psychology, sociology, etc., researchers are presently exploring the flavor compounds that are absent in modern tomatoes, analyzing the reasons for these losses, studying the mechanisms of flavor formation, determining more scientifically valid and reasonable methods to evaluate flavor, clarifying the road map of tomato flavor improvement, and cultivating some tomato varieties with excellent flavor.

## Conclusion

The flavor of 71 tomato accessions have significant differences, which can be divided into four categories according to the flavor evaluation scores. Seventy-one tomato accessions can be divided into four classes: among these, accessions PL11, PC4, PC2, PC8, RL35, RC6, and RC10 had better flavor; accessions PC4, PC8, RC10, RL2, and RL35 had better tomato taste; and accessions PL11, PC2, and RC6 had better tomato odor. The important flavor compounds were soluble solids, fructose, glucose, citric acid, the sugar and acid ratio, 1-hexanol, (*Z*)-3-hexenol, 2-phenylethanol, hexanal, (*E*)-2-hexenal, (*E*)-2-heptenal, (*E,E*)-2,4-decadienal, 2,6,6-timethyl-1-cyclohexene-1-carboxaldehyde, 6-methyl-5-hepten-2-one, (*E*)-6,10-dimetyl-5,9-undecadien-2-one, 4-(2,6,6-trimethyl-1-cyclohexen-1-yl)-3-buten-2-one, and 2-isobutylthiazole. These chemicals were positively correlated with flavor preferences and can, therefore, be used as targets for flavor improvement.

## Data Availability Statement

All data generated or analyzed during this study are included in this published article and its Supplementary Material. The mass spectronomy data can be found here: doi: 10.6084/m9.figshare.12744695.

## Ethics Statement

The studies involving human participants were reviewed and approved by the Northwest A&F University Institutional Review Board. The patients/participants provided their written informed consent to participate in this study.

## Author Contributions

YL: conceptualization, resources, supervision, writing—review and editing, and project administration. GC: conceptualization, performing the experiments, data analysis, and writing—original draft preparation. FZ: supervision and writing—review and editing. PC: methodology, date analysis, and formula analysis. LW: visualization. YS: data analysis; AE-S: writing—review and editing. All authors contributed to the article and approved the submitted version.

## Conflict of Interest

The authors declare that the research was conducted in the absence of any commercial or financial relationships that could be construed as a potential conflict of interest.

## Footnotes

1 http://www.heatmapper.ca/

## Supplementary Material

Supplementary Table 1 Concentrations profiles of taste compounds of mature tomato fruits. ^*a*^In calibration curve (*y* = kx + b), “y” represents the theoretical concentration of the labeled standard, and “x” represents the peak area ratio of a compound in the internal standard. ^*b*^‘—’ indicates that a labeled standard does not exist and, thus, a calibration curve was could not be created.

Supplementary Table 2 Concentrations of volatiles of different functional groups in 71 tomato accessions (10^–9^ kg L^–1^). n.d. represents not detected.

Supplementary Table 3 Concentrations of volatiles from different metabolic precursors in 71 tomato accessions (10^–9^ kg L^–1^). n.d. represents not detected.

Supplementary Table 4 Hedonism scores and odor activity values in 71 tomato accessions. ^*a*^PC, PL, RC, and RL indicated pink cherry tomato, pink large-fruited tomato, red cherry tomato and red large-fruited tomato, respectively. ^*b*^C_*i*_ values represent the scores of the DTOPSIS analysis.

## Abbreviations

PC: pink cherry tomato
PL: large-fruited tomato
RC: red cherry tomato
RL: red large-fruited tomato
RT: retention time
RI: retention index
HPLC: high-performance liquid chromatography
HS-SPME: headspace solid-phase microextraction
GC-O-MS: gas chromatography–olfactometry–mass spectrometry
SD: standard deviations
CV: coefficient of variations
OAV: odor activity values.
