# Supplementary material for: Comparing the Flavor Characteristics of 71 Tomato (Solanum lycopersicum) Accessions in Central Shaanxi
Source: Front Plant Sci. 2020 Dec 10;11:586834. doi: 10.3389/fpls.2020.586834 (PMC7758415; doi:10.3389/fpls.2020.586834)
Supplement: Supplementary Table 1 — Concentrations profiles of taste compounds of mature tomato fruits. aIn calibration curve (y = kx + b), “y” represents the theoretical concentration of the labeled standard, and “x” represents the peak area ratio of a compound in the internal standard. b‘—’ indicates that a labeled standard does not exist and, thus, a calibration curve was could not be created. [file Table_1.DOCX]

**Table S1. Concentrations profiles of taste compounds of mature tomato fruits.**

| **Taste compound** | **Calibration curve**^a^ | **Regression coefficient (R**2) | **Concentration range** | **Average** | **Variation coefficient** |
| --- | --- | --- | --- | --- | --- |
| **Soluble solids (%)** | —^b^ | — | 3.67—11.43 | 6.013 | 0.26 |
| **Fructose (mg 100 g**^-1^**)** | y=2.856x-0.320 | 0.994 | 910—2400 | 1462 | 0.25 |
| **Glucose (mg 100 g**^-1^**)** | y=2.734x-0.318 | 0.996 | 560—1600 | 911 | 0.25 |
| **Citric acid (mg 100 g**^-1^**)** | y=11.308x-0.152 | 0.991 | 120—540 | 281 | 0.31 |
| **Malic acid (mg 100 g**^-1^**)** | y=8.308x-0.125 | 0.990 | 60-390 | 159 | 0.36 |
| **Sugar and acid ratio** | — | — | 2.77—10.89 | 5.716 | 0.34 |

^a^ In calibration curve (y = kx + b), “y” represents the theoretical concentration of the labeled standard, and “x” represents the peak area ratio of a compound in the internal standard (Wang and Seymour, 2017).

^b^ ‘—’ indicates that a labeled standard does not exist and, thus, a calibration curve was could not be created.
