# Supplementary material for: Comparing the Flavor Characteristics of 71 Tomato (Solanum lycopersicum) Accessions in Central Shaanxi
Source: Front Plant Sci. 2020 Dec 10;11:586834. doi: 10.3389/fpls.2020.586834 (PMC7758415; doi:10.3389/fpls.2020.586834)
Supplement: Supplementary Table 4 — Hedonism scores and odor activity values in 71 tomato accessions. aPC, PL, RC, and RL indicated pink cherry tomato, pink large-fruited tomato, red cherry tomato and red large-fruited tomato, respectively. bCi values represent the scores of the DTOPSIS analysis. [file Table_4.DOCX]

**Table S4. Hedonism scores and odor activity values in 71 tomato accessions.**

| **Class** | **Accession** | **Sweetness** | **Sourness** | **Sweetness/sourness** | **Tomato-like flavor** | **Overall acceptability** | **C_i(taste)_^b^** | **Floral** | **Green** | **Vegetable-like** | **Fruity** | **Fatty** | **Irritation** | **C_i(odor)_** | **C_i(overall)_** | **Ranking** |
| --- | --- | --- | --- | --- | --- | --- | --- | --- | --- | --- | --- | --- | --- | --- | --- | --- |
| Ⅰ | **PL11^a^** | 4.43 | 4 | 1.11 | 4.71 | 5.13 | -0.15 | 99 | 574 | 80 | 227 | 64 | 153 | 6.85 | 6.7 | 1 |
|  | **PC4** | 8 | 2.25 | 3.56 | 7.75 | 8 | 4.45 | 27 | 26 | 28 | 22 | 33 | 33 | 0.2 | 4.64 | 2 |
|  | **PC2** | 6.71 | 4.29 | 1.56 | 6.14 | 6.63 | 1.03 | 132 | 186 | 51 | 75 | 178 | 101 | 2.37 | 3.4 | 3 |
|  | **RL2** | 6.75 | 5 | 1.35 | 7.75 | 7.75 | 1.75 | 25 | 53 | 24 | 42 | 33 | 60 | 0.86 | 2.62 | 4 |
|  | **PC8** | 7.75 | 5 | 1.55 | 7.75 | 7.9 | 2.08 | 30 | 127 | 36 | 46 | 64 | n.d. | 0.03 | 2.11 | 5 |
|  | **RL35** | 5.5 | 5.5 | 1 | 7.25 | 7.25 | 1.36 | 32 | 141 | 25 | 39 | 27 | 34 | 0.2 | 1.56 | 6 |
|  | **RC6** | 3.5 | 3.71 | 0.94 | 5.14 | 5.63 | -0.35 | 175 | 167 | 37 | 258 | 79 | 82 | 1.86 | 1.52 | 7 |
|  | **RC10** | 7.25 | 5.75 | 1.26 | 7.25 | 7.75 | 2.08 | 11 | 30 | 7 | 21 | 8 | 3 | -0.64 | 1.44 | 8 |
|  | **PL13** | 4.86 | 4.57 | 1.06 | 5 | 6.3 | 0.38 | 60 | 210 | 73 | 100 | 65 | 42 | 0.7 | 1.07 | 9 |
|  | **PC7** | 5.86 | 3.86 | 1.52 | 4.43 | 6.57 | 0.18 | 60 | 288 | 53 | 95 | 69 | 42 | 0.86 | 1.04 | 10 |
| Ⅱ | **RL33** | 5.5 | 5.5 | 1 | 7.25 | 6.65 | 1.29 | 32 | 53 | 20 | 30 | 23 | 5 | -0.47 | 0.82 | 11 |
|  | **RL19** | 5.75 | 3.5 | 1.64 | 5.5 | 5.25 | -0.08 | 64 | 318 | 94 | 139 | 184 | 32 | 0.86 | 0.78 | 12 |
|  | **PL7** | 4 | 5.57 | 0.72 | 6 | 5.63 | 0.7 | 45 | 434 | 138 | 149 | 101 | 13 | 0.03 | 0.73 | 13 |
|  | **PL9** | 2.75 | 6 | 0.46 | 6 | 6 | 0.57 | 52 | 58 | 24 | 54 | 48 | 26 | 0.03 | 0.6 | 14 |
|  | **PL4** | 4.86 | 5.29 | 0.92 | 5.29 | 6 | 0.7 | 43 | 57 | 51 | 52 | 125 | 17 | -0.14 | 0.57 | 15 |
|  | **RC8** | 4.86 | 5.14 | 0.95 | 5.43 | 6 | 0.7 | 34 | 361 | 26 | 50 | 23 | 13 | -0.14 | 0.57 | 16 |
|  | **PC3** | 6.71 | 4.29 | 1.56 | 6.14 | 6.63 | 1.03 | 18 | 137 | 16 | 28 | 26 | 5 | -0.47 | 0.56 | 17 |
|  | **RC9** | 6.71 | 2.86 | 2.35 | 6.71 | 7.29 | 0.51 | 58 | 141 | 194 | 44 | 64 | 25 | 0.03 | 0.54 | 18 |
|  | **PC1** | 6.86 | 3.86 | 1.78 | 6.43 | 6.38 | 0.84 | 30 | 77 | 20 | 27 | 50 | 14 | -0.31 | 0.53 | 19 |
|  | **RL20** | 4.75 | 4.75 | 1 | 5.5 | 5.75 | 0.44 | 55 | 23 | 20 | 17 | 38 | 26 | 0.03 | 0.47 | 20 |
|  | **RL12** | 2.57 | 5.86 | 0.44 | 3.71 | 4.63 | 0.24 | 43 | 157 | 42 | 57 | 40 | 27 | 0.2 | 0.44 | 21 |
|  | **RL36** | 5.25 | 5.25 | 1 | 5.25 | 5.5 | 0.7 | 23 | 97 | 25 | 49 | 37 | 13 | -0.31 | 0.4 | 22 |
|  | **PC6** | 6.14 | 3.29 | 1.87 | 5.86 | 7.38 | 0.31 | 29 | 368 | 54 | 50 | 88 | 16 | 0.03 | 0.34 | 23 |
|  | **RC1** | 3 | 5.29 | 0.57 | 4.71 | 3.88 | 0.11 | 22 | 46 | 12 | 35 | 14 | 33 | 0.2 | 0.31 | 24 |
|  | **RL28** | 5 | 4.43 | 1.13 | 5.29 | 4 | 0.11 | 22 | 48 | 17 | 25 | 32 | 34 | 0.2 | 0.31 | 25 |
|  | **RL26** | 3 | 5.75 | 0.52 | 4.25 | 4.75 | 0.31 | 5 | 11 | 7 | 50 | 8 | n.d. | -0.14 | 0.17 | 26 |
|  | **RC7** | 6 | 3.57 | 1.68 | 6.57 | 7.38 | 0.57 | 11 | 65 | 18 | 16 | 30 | 11 | -0.47 | 0.1 | 27 |
|  | **PL6** | 3.86 | 3.86 | 1 | 4.29 | 5.4 | -0.28 | 36 | 135 | 26 | 46 | 43 | 36 | 0.36 | 0.08 | 28 |
|  | **RC5** | 4.57 | 5 | 0.91 | 4.14 | 6.26 | 0.38 | 38 | 84 | 24 | 59 | 42 | 13 | -0.31 | 0.07 | 29 |
|  | **RL8** | 3.14 | 4.86 | 0.65 | 4.29 | 4.38 | -0.02 | 17 | 26 | 15 | 22 | 38 | 26 | 0.03 | 0.01 | 30 |
| Ⅲ | **RL23** | 3 | 2.75 | 1.09 | 2.75 | 1.75 | -1.33 | 126 | 188 | 55 | 129 | 52 | 55 | 1.2 | -0.13 | 31 |
|  | **RL37** | 4 | 5 | 0.8 | 6.25 | 2.25 | -0.02 | 68 | 97 | 30 | 87 | 42 | 18 | -0.14 | -0.16 | 32 |
|  | **RC4** | 3.14 | 5.14 | 0.61 | 6.29 | 5.38 | 0.31 | 22 | 104 | 21 | 43 | 34 | 5 | -0.47 | -0.16 | 33 |
|  | **RL25** | 3.43 | 5.43 | 0.63 | 4.43 | 4.88 | 0.31 | 13 | 52 | 17 | 22 | 18 | 6 | -0.47 | -0.16 | 34 |
|  | **RL27** | 4.5 | 5 | 0.9 | 4.5 | 5.25 | 0.31 | 33 | 103 | 24 | 64 | 55 | 9 | -0.47 | -0.16 | 35 |
|  | **RL1** | 5 | 4.75 | 1.05 | 5.25 | 5.25 | 0.44 | 24 | 53 | 14 | 37 | 37 | 4 | -0.64 | -0.2 | 36 |
|  | **PL2** | 4.75 | 3.25 | 1.46 | 6.25 | 5.75 | -0.28 | 24 | 22 | 24 | 76 | 60 | 31 | 0.03 | -0.25 | 37 |
|  | **PL1** | 5.75 | 3.75 | 1.53 | 6 | 6.25 | 0.38 | 23 | 16 | 14 | 36 | 38 | 2 | -0.64 | -0.26 | 38 |
|  | **RL3** | 2.57 | 5.86 | 0.44 | 4.86 | 5 | 0.38 | 8 | 21 | 6 | 14 | 4 | 2 | -0.64 | -0.26 | 39 |
|  | **RL9** | 3.14 | 5.14 | 0.61 | 4.86 | 5 | 0.18 | 66 | 95 | 45 | 46 | 74 | 10 | -0.47 | -0.29 | 40 |
|  | **RC2** | 4.57 | 4.43 | 1.03 | 5.57 | 5.85 | 0.31 | 17 | 46 | 16 | 28 | 23 | 2 | -0.64 | -0.33 | 41 |
|  | **RL13** | 2.75 | 2.75 | 1 | 4.5 | 2.75 | -1.2 | 42 | 71 | 34 | 16 | 34 | 61 | 0.86 | -0.34 | 42 |
|  | **RL31** | 3.5 | 4 | 0.88 | 5 | 5 | -0.28 | 24 | 99 | 29 | 55 | 32 | n.d. | -0.14 | -0.42 | 43 |
|  | **RL14** | 4.5 | 3.5 | 1.29 | 5.5 | 3.75 | -0.48 | 29 | 78 | 23 | 45 | 33 | 28 | 0.03 | -0.45 | 44 |
|  | **PL12** | 3.5 | 4 | 0.88 | 6.25 | 5.25 | -0.15 | 40 | 126 | 29 | 51 | 22 | 10 | -0.31 | -0.46 | 45 |
|  | **PL3** | 5.5 | 2.75 | 2 | 6.5 | 6.5 | -0.35 | 15 | 55 | 17 | 30 | 23 | n.d. | -0.14 | -0.49 | 46 |
|  | **PL15** | 2.14 | 4.29 | 0.5 | 2.86 | 3.25 | -0.54 | 19 | 41 | 24 | 30 | 28 | 28 | 0.03 | -0.52 | 47 |
|  | **RL16** | 5.14 | 3.43 | 1.5 | 5.29 | 5.5 | -0.22 | 11 | 58 | 22 | 4 | 8 | 17 | -0.31 | -0.52 | 48 |
|  | **RL6** | 3.25 | 4.5 | 0.72 | 4.25 | 2.25 | -0.35 | 14 | 12 | 7 | 18 | 23 | 19 | -0.31 | -0.65 | 49 |
|  | **RC11** | 5.86 | 2.71 | 2.16 | 5.43 | 6 | -0.54 | 11 | 25 | 15 | 25 | 40 | 20 | -0.14 | -0.68 | 50 |
|  | **RL21** | 5.75 | 3.5 | 1.64 | 5.5 | 5.25 | -0.08 | 62 | 13 | 62 | 14 | 83 | 2 | -0.64 | -0.72 | 51 |
|  | **PC5** | 2.43 | 4.71 | 0.52 | 4 | 4.71 | -0.15 | 11 | 91 | 9 | 41 | 13 | 2 | -0.64 | -0.79 | 52 |
|  | **RL10** | 3.25 | 4.5 | 0.72 | 4.25 | 2.25 | -0.35 | 34 | 40 | 18 | 46 | 28 | 6 | -0.47 | -0.82 | 53 |
|  | **PL5** | 4.43 | 3.29 | 1.35 | 5.86 | 5.38 | -0.41 | 26 | 70 | 37 | 68 | 56 | 10 | -0.47 | -0.88 | 54 |
|  | **PL14** | 4.25 | 3.5 | 1.21 | 3.25 | 4.5 | -0.61 | 26 | 32 | 30 | 41 | 33 | 13 | -0.31 | -0.91 | 55 |
|  | **RC3** | 3.57 | 4 | 0.89 | 4.57 | 4.88 | -0.28 | 22 | 24 | 10 | 36 | 27 | 2 | -0.64 | -0.92 | 56 |
|  | **PL8** | 3.25 | 3 | 1.08 | 3 | 2.25 | -1.13 | 37 | 47 | 30 | 112 | 22 | 33 | 0.2 | -0.94 | 57 |
|  | **RL24** | 5.5 | 3 | 1.83 | 5.5 | 5 | -0.48 | 61 | 27 | 22 | 67 | 55 | 9 | -0.47 | -0.95 | 58 |
| Ⅳ | **RL7** | 2.5 | 4 | 0.63 | 4.25 | 3.75 | -0.54 | 30 | 81 | 23 | 36 | 50 | 6 | -0.47 | -1.02 | 59 |
|  | **RL15** | 2.86 | 4.14 | 0.69 | 2.86 | 3.13 | -0.54 | 10 | 64 | 11 | 20 | 11 | 10 | -0.47 | -1.02 | 60 |
|  | **RL4** | 4.75 | 2.75 | 1.73 | 5.25 | 4 | -0.94 | 19 | 17 | 11 | 31 | 21 | 20 | -0.14 | -1.08 | 61 |
|  | **PL10** | 5 | 2.86 | 1.75 | 4.43 | 5.38 | -0.74 | 17 | 80 | 40 | 68 | 49 | 5 | -0.47 | -1.21 | 62 |
|  | **RL17** | 2 | 3.29 | 0.61 | 2.29 | 2.5 | -1.07 | 30 | 119 | 30 | 28 | 16 | 16 | -0.31 | -1.37 | 63 |
|  | **RL22** | 3.5 | 2.75 | 1.27 | 2.75 | 2.5 | -1.27 | 22 | 28 | 12 | 39 | 13 | 22 | -0.14 | -1.4 | 64 |
|  | **RL32** | 2.75 | 3.25 | 0.85 | 2.75 | 2.75 | -1 | 17 | 37 | 15 | 23 | 24 | 9 | -0.47 | -1.48 | 65 |
|  | **RL5** | 2.43 | 3 | 0.81 | 2.86 | 3.13 | -1.13 | 18 | 31 | 11 | 16 | 18 | 11 | -0.47 | -1.61 | 66 |
|  | **RL29** | 2.86 | 2.43 | 1.18 | 5.86 | 4.75 | -1.27 | 24 | 121 | 23 | 47 | 45 | 6 | -0.47 | -1.74 | 67 |
|  | **RL11** | 2 | 2.43 | 0.82 | 4.14 | 2.25 | -1.46 | 16 | 17 | 10 | 22 | 17 | 7 | -0.47 | -1.94 | 68 |
|  | **RL30** | 2.57 | 2.14 | 1.2 | 2 | 2.25 | -1.66 | 23 | 37 | 18 | 33 | 20 | 17 | -0.31 | -1.97 | 69 |
|  | **RL18** | 2.57 | 2.14 | 1.2 | 2 | 2.25 | -1.66 | 31 | 54 | 21 | 23 | 32 | 6 | -0.47 | -2.13 | 70 |
|  | **RL34** | 3.5 | 1.5 | 2.33 | 3.5 | 3.25 | -2.05 | 30 | 40 | 13 | 39 | 6 | 2 | -0.64 | -2.69 | 71 |

^a^ PC, PL, RC, and RL indicated pink cherry tomato, pink large-fruited tomato, red cherry tomato, and red large-fruited tomato, respectively.

^b^ C_i_ values represent the scores of the DTOPSIS analysis.
